# Pulmonary artery pressure is associated with extracellular volume fraction in patients with normal left ventricular function

**DOI:** 10.1186/1532-429X-18-S1-P120

**Published:** 2016-01-27

**Authors:** George M Cater, Erik B Schelbert, Marc Simon, Peter Kellman, Timothy C Wong

**Affiliations:** 1 Heart and Vascular Institute, University of Pittsburgh Medical Center, Pittsburgh, PA USA; 2 National Heart, Lung, and Blood Institute, National Institutes of Health, Bethesda, MD USA

## Background

Elevated pulmonary artery pressures (PAP) from type II pulmonary hypertension (PH) is caused by a heterogeneous group of disorders. Myocardial fibrosis (MF) is a common pathological finding in many cardiovascular diseases; however its relationship with PAP is not well characterized. In particular, among patients with heart failure and preserved ejection fraction (HFpEF), MF may be a pathophysiologic mechanism contributing to disease severity and poor outcomes. This paradigm would be strengthened if MF were demonstrated to independently predict PAP in HFpEF. Cardiac MRI techniques quantifying extracellular volume matrix (ECV), are well validated markers of MF and may provide more comprehensive assessment over traditional late gadolinium enhancement (LGE) techniques. Thus, the goal of this study was to demonstrate that ECV is an independent predictor of PAP, particularly in patients with preserved left ventricular ejection fraction (EF).

## Methods

We assessed 276 patients without infiltrative cardiac disorders, type I, III, IV PH or severe valvular disease, referred for clinical, contrast enhanced 1.5T cardiac MR and contemporaneous echocardiogram within 7 days. PAP was estimated by Doppler interrogation of tricuspid regurgitation. ECV measures were obtained using pre and post contrast myocardial and blood T1, adjusting for hematocrit. Univariable and multivariable forward linear regression assessed predictors of PAP. Analysis was stratified by EF with threshold of ≥50% (n = 147) as normal.

## Results

In our cohort, 60% were male; median PAP was 33 mmHg (range: 10-68); median ECV was 29.6% (range: 19-44). For each 3% increase in ECV, PAP increased by 2 mmHg (R^2^ = 0.08, p < 0.01) in univariable analysis. In stepwise multivariable analysis of 20 clinical and imaging factors, left atrial volume index (LAVI), right ventricular (RV) systolic dysfunction, RV enlargement, E/e', age and ECV were significant predictors of PAP (R^2^ = 0.3, p < 0.01). In stratified univariate analysis, ECV correlation to PAP was strongest among patients with preserved EF (R^2^ = 0.13, p < 0.01), but was not significant for those with decreased EF (R^2^ = 0.01, p = 0.28). Multivariable regression demonstrated age, ECV, RV enlargement, E/e' and LAVI to be independent predictors of PAP in preserved EF (R^2^ = 0.35, p < 0.01). Age, E/A, RV systolic dysfunction and LAVI were predictors of PAP in EF < 50% (R^2^ = 0.25, p < 0.01), but not ECV. In multivariable analysis, LGE was not significantly associated with PAP.

## Conclusions

Etiologies of elevated PAP in patients with type II PH are heterogeneous. ECV is an independent predictor of PAP only in the normal EF group despite extensive factor adjustment, suggesting that MF may contribute to elevated left ventricular filling pressures and correspondingly, elevated PAP. Although the strength of association between PAP and ECV was modest, the persistence of ECV in the final preserved EF model suggests that MF may represent a key mechanistic contributor to myocardial dysfunction in this population.

